# Cultural adaptations of third-wave psychotherapies in Gulf Cooperation Council countries: A systematic review

**DOI:** 10.1177/13634615241227691

**Published:** 2024-02-08

**Authors:** Duaa H. Alrashdi, Aisha H. Alyafei, Samar A. Alanazi, Carly Meyer, Rebecca L. Gould

**Affiliations:** 1 Division of Psychiatry, Faculty of Brain Sciences, 4919 University College London, UK; 2 Department of Health Sciences, Faculty of Health and Rehabilitation Sciences, 112893 Princess Nourah bint Abdulrahman University, KSA; 3 Prince Mohammed Bin Salman Center for Autism and Developmental Disorders, 37853 Prince Sultan Military Medical City, KSA; 4 School of Health and Rehabilitation Sciences, 1974 The University of Queensland, Australia; 5 Department of Clinical, Educational, and Health Psychology, Faculty of Brain Sciences, 4919 University College London, UK

**Keywords:** culture, Gulf Cooperation Council, mental health, systematic review, third-wave psychotherapies

## Abstract

The effectiveness of third-wave psychotherapies has been demonstrated in a range of mental and physical health conditions in Western cultures. However, little is known about the cultural appropriateness and effectiveness of third-wave psychotherapies for Gulf Cooperation Council (GCC) populations. This review aimed to critically evaluate cultural adaptations to third-wave psychotherapies and explored the effectiveness of these interventions on physical and mental health outcomes in GCC populations. Five bibliographic databases and grey literature were searched; both English and Arabic studies conducted in the GCC were included. Mental and physical health-related outcomes were included. Eleven studies were identified. The overall degree of cultural adaptation ranged from 2 to 5, based on Bernal et al.'s cultural adaptation framework. Language and assessment tools were most frequently adapted. Several studies incorporated goal, method, and context adaptations, whereas metaphor and content were least frequently adapted. None of the studies incorporated person or concept adaptations. Culturally adapted third-wave psychotherapies were associated with improvement in numerous mental health outcomes, including psychological distress, well-being, and psychological traits. No physical health outcomes were identified. Although findings are promising with respect to the effectiveness of third-wave psychotherapies for GCC populations, they should be interpreted with caution due to the small number of studies conducted, cultural adaptation evaluations relying on explicit reporting in studies, and the weak methodological quality of studies. Future rigorous research is needed in the evaluation of culturally adapted third-wave psychotherapies in GCC populations, with more comprehensive reporting of cultural considerations.

## Introduction

The effectiveness of third-wave psychotherapies has been demonstrated in a range of mental and physical health conditions, including anxiety, depression, and chronic pain (Churchill et al., 2013; Gloster et al., 2020; Makris & Dorstyn, 2022). Third-wave psychotherapies emphasise experiential rather than didactic means of learning, with an underlying philosophy of being contextualistic rather than mechanistic; therefore, they incorporate different strategies, including both mindfulness and acceptance, to help people construct flexible and effective behavioural repertoires (Hayes, 2004). Through these means, third-wave psychotherapies focus on a person's relationship to their internal experiences, such as thoughts and emotions, rather than trying to change these experiences, as is the case in second-wave psychotherapies (e.g., cognitive behavioural therapy) (Hayes & Hofmann, 2017). Examples of third-wave psychotherapies include acceptance and commitment therapy (ACT), mindfulness-based interventions (MBIs) such as mindfulness-based stress reduction (MBSR) and mindfulness-based cognitive therapy (MBCT), dialectical behavioural therapy (DBT), and compassion-based interventions (CBIs) such as compassion-focused therapy.

Despite the growing interest in third-wave psychotherapies, much of the previous research has examined their effectiveness in a narrow range of populations only. For example, a retrospective systematic review found that 79% of participants in 94 randomised controlled trials (RCTs) of MBIs self-identified as White (Eichel et al., 2021). A critical criterion for assessing an intervention's effectiveness is its cultural appropriateness for the target population (Bernal & Sáez-Santiago, 2006). The cultural applicability of third-wave psychotherapies to Gulf Cooperation Council (GCC) populations remains unclear. This highlights a major gap in the third-wave psychotherapies literature given that the GCC comprises more than 57 million people who share similar regulations, systems, cultures, and customs: namely, the Kingdom of Saudi Arabia, State of Kuwait, State of Qatar, Kingdom of Bahrain, Sultanate of Oman, and United Arab Emirates (GCC, 2022; GCC-STAT, 2020).

Cultural adaptation is “the systematic modification of an evidence-based treatment (EBT) or intervention protocol to consider language, culture, and context in such a way that it is compatible with the client's cultural patterns, meanings, and values” (Bernal et al., 2009, p. 362). A framework of cultural adaptation for psychological interventions has been developed by Bernal and colleagues (Bernal & Sáez-Santiago, 2006; Bernal et al., 1995), which comprises eight dimensions: language, person, metaphor, content, goals, concept, method, and context (see Table 1). It is important to culturally adapt psychological interventions because it could improve the appropriateness, efficacy, and implementation of EBTs to specific populations (Castellanos et al., 2020; Lau, 2006; Shehadeh et al., 2016). For example, Gearing et al. (2013) reported that the most commonly reported barrier to implementing psychosocial and mental health interventions in Arab countries is related to aspects of cultural context, such as beliefs, values, and stigmas.

**Table 1.** Definitions and associated criteria based on Bernal et al.'s eight dimensions of cultural adaptations.

Dimension	Bernal et al.'s definition	Definition as applied to this systematic review	Criterion
**Language**	“Culturally appropriate; culturally syntonic language.”	Culturally appropriate language for the target population. Since included studies were conducted in GCC countries, the Arabic language should not be necessarily explicitly stated. The intervention language was considered to presumably be Arabic— unless otherwise reported.	Was the intervention delivered in and/or translated to Arabic?
**Person**	“Role of ethnic/racial similarities and differences between client and therapist in shaping therapy relationship.”	Culturally appropriate consideration of patient–therapist similarities and differences. The therapist's ethnicity should be explicitly stated as being Arab to be coded as having the adaptation.	Was there any cultural consideration of the patient–therapist similarities and differences?
**Metaphor**	“Symbols and concepts shared with the population; sayings or concepts in treatment.”	Sayings, stories, poets, examples, wisdom, and symbols used in the intervention were adapted to fit within GCC culture.	Was metaphor considered in the cultural adaptation of the intervention?
**Content**	“Cultural knowledge: values, costumes and traditions; uniqueness of groups (social, economic, historical, political).” “Refers to cultural knowledge about values, customs, and traditions shared by ethnic and minority groups. Cultural and ethnic uniqueness should be integrated into all phases of a treatment process, including assessment and treatment planning.”	Adaptation of intervention contents that have been made and informed by the knowledge of the GCC culture, including assessment tools and intervention plan.	Did the contents of the intervention adapt to the local culture (e.g., incorporating relevant cultural concepts or materials into the intervention manual, and focusing on the population's values? Did the assessment tools of outcomes adapt to the local culture?
**Goals**	“Transmission of positive and adaptive cultural values; support adaptive values from the culture of origin.”	The discussion and agreement of intervention goals between therapist and client and/or the consistency of intervention goals with population values, traditions, and customs.	Were intervention goals discussed with clients and did they match with their values, tradition, and customs? Did intervention goals consider in relation to cultural knowledge?
**Concept**	“Treatment concepts consonant with culture and context: dependence vs. interdependence vs. independence, emic (within culture, particular) over etic (outside culture, universal).”	Integration of cultural aspects into the conceptualisation of intervention model and/or presenting client distress and/or formatting process of change.	Did the intervention adapt to consider the conceptualisation of psychological theory / presenting distress / intervention process of change (e.g., social roles, beliefs about the problem)?
**Method**	“Development and/or cultural adaptation of treatment methods.”	Practical aspects of delivering intervention which were informed by the knowledge of the population and/or GCC culture that were distinct from therapeutic aspects.	Did the method of delivery adapt to the culture (e.g., access to facilities)?
**Context**	“Consideration of changing contexts in assessment during treatment or intervention: acculturative stress, phase of migration, developmental stage, social support and relationship to country of origin, economic and social context of intervention.”	Consideration and/or discussion of the broader social, economic, and political aspects of the participants that were not directly the target or goals of the intervention.	Did the intervention adapt to the broader social, economic, or political context (e.g., consideration of accessibility, feasibility, and acceptability of the intervention)?

*Sources*. Bernal et al. (1995, p. 74); Bernal & Sáez-Santiago (2006, p. 128); GCC = Gulf Cooperation Council.

Previous studies have demonstrated the acceptability of culturally adapted third-wave psychotherapies among the Arab population. For example, Arabic-speaking communities in Australia showed a statistically significant reduction in psychological distress, such as depression, anxiety, and stress, after receiving a culturally adapted MBI at post-intervention and 12-week follow-up (Blignault et al., 2019). Similarly, a culturally adapted MBI showed a statistically significant improvement in stress, depression, anxiety, and mindfulness among Arab parents of children with autism spectrum disorder in Jordan compared to treatment as usual at post-intervention (Rayan & Ahmad, 2017). These programmes were adapted to enhance cultural sensitivity for the Arab population, for example by introducing mindfulness in a more relevant manner using the Arabic terms “*Tafakkur*” or “*alhudur althihni*,” which have similar meanings to mindfulness. Consequently, this suggests that third-wave psychotherapies are acceptable and effective for implementation and are culturally appropriate for use within different contexts among Arab populations.

Furthermore, meta-analytic evidence suggests that culturally adapted psychological interventions are more effective at improving mental health outcomes than non-culturally adapted interventions. For example, one meta-analysis that included 78 studies with a 95% non-European American sample found that culturally adapted psychological interventions were more effective at improving mood, anxiety, and psychotic symptoms in comparison to non-culturally adapted versions of the same interventions (Hall et al., 2016). Another meta-analysis of eight studies of culturally adapted, minimally guided psychological interventions reported a positive relationship between the number of cultural adaptations and effect sizes for depression and anxiety outcomes, whereby effect sizes increased with the number of Bernal et al.'s cultural adaptation dimensions implemented (Shehadeh et al., 2016). However, in both meta-analyses, none of the samples were from the GCC, and only one study of a third-wave psychotherapy was included.

To the authors’ knowledge, no review to date has examined the cultural appropriateness or effectiveness of third-wave psychotherapies for GCC populations, despite the uniqueness of their culture. It has been emphasised that Arab culture is highly diverse, stressing the need to explore the distinct differences within the Arab population when addressing the adaptation of mental health interventions (Gearing et al., 2013). For example, Algahtani et al. (2017) emphasised that therapists must be aware of subcultural variations within Saudi societies, such as the roles of family and gender. Consequently, this review aimed to: (a) systematically review and critically evaluate cultural adaptations of third-wave psychotherapies for GCC populations using Bernal et al.'s cultural adaptation framework; and (b) explore the effectiveness of culturally adapted third-wave psychotherapies for mental and physical health outcomes in these populations in comparison to both control conditions and non-culturally adapted third-wave psychotherapies.

## Method

### Eligibility criteria

Studies were included based on the following criteria: (1) they must have been RCTs, non-randomised controlled trials (CTs), or pre-post cohort studies; (2) published or unpublished studies; (3) the studies must have been conducted in GCC countries (i.e., Kingdom of Saudi Arabia, State of Kuwait, State of Qatar, Kingdom of Bahrain, Sultanate of Oman, and United Arab Emirates); (4) the studies examined mental health outcomes (i.e., any outcomes or traits related to mental or psychological health, such as depression, mindfulness traits, etc.) and/or physical health outcomes (e.g., pain, eating/sleeping-related behaviours) that were assessed using appropriate measures (e.g., self-report questionnaires, clinician evaluations); (5) participants were aged ≥ 17 years old (to accommodate global variations in the age at which students typically start university); and (6) the intervention was based on a third-wave psychotherapy. Third-wave psychotherapy was defined as any intervention drawing from mindfulness and/or acceptance strategies that aims to help clients construct flexible and effective behaviour repertories (Hayes, 2004)—namely, ACT, MBIs, DBT, and CBIs.

Studies were excluded based on the following criteria: (1) they were case-control studies or case reports, cross-sectional studies, qualitative studies, study protocols, systematic reviews and meta-analyses, or meeting abstracts; (2) the intervention only comprised a small component of a third-wave psychotherapy or was not consistent with third-wave psychotherapy principles (e.g., yoga, tai chi, transcendental meditation); (3) third-wave psychotherapy was combined with other psychological treatments (e.g., cognitive behavioural therapy); and (4) the studies were published in a language other than English or Arabic.

### Search strategy

Four electronic databases (PubMed, Web of Science, PsycInfo, and Embase), one Arabic electronic database (Almandumah, accessed via the Saudi Digital Library), and grey literature (via ProQuest) were searched separately from the date of inception to 16–23 May 2022. English and Arabic search terms were developed based on key concepts related to third-wave psychotherapies, the GCC, Arabs, and Islam (see Appendix A; supplementary materials). There were no specific search limitations or restrictions. Reference lists of included and relevant studies were searched manually.

### Selection process and data collection

After duplicates were removed, titles and abstracts were screened blindly and independently by two researchers (DA and AA), except for the Almandumah database, which was screened by one researcher (DA) due to the difficulty of importing search results into any management platform. Full-text articles were retrieved for potentially relevant studies and assessed independently and blindly against eligibility criteria by two researchers (DA and AA). A standardised data extraction form was created, and data on participants, interventions, outcomes, and results were extracted. Study authors were contacted to provide clarification about queries, where necessary. Data extraction was conducted independently and blindly by two researchers (DA and SA). Disagreements were resolved via discussion with two other researchers (RG and CM).

### Evaluation of cultural adaptation

Bernal et al.'s cultural adaptation framework was used to evaluate the degree of cultural adaptations of third-wave psychotherapies. To facilitate this, following procedures employed in previous reviews of culturally adapted psychological interventions (Castellanos et al., 2020; Shehadeh et al., 2016), a definition of each dimension was created based on Bernal et al.'s eight dimensions of cultural adaptations (see Table 1). These definitions were used as standard criteria to critically evaluate the cultural adaptations. Each study was assigned a score according to the number of dimensions completed. For the content dimension, Bernal and Sáez-Santiago (2006) emphasised that cultural uniqueness should be integrated into all phases of the intervention process in both the assessment and intervention plans. Therefore, the adaptation of assessment tools and intervention content was evaluated separately; consequently, scores on the cultural adaptation scale ranged from 0 to 9, with a higher score indicating a greater degree of cultural adaptation. Two bilingual (English- and Arabic-speaking) coders (DA and SA) evaluated the degree of cultural adaptation in each study independently. Disagreements were resolved via discussion with two other researchers (RG and CM).

### Quality assessment

The Quality Assessment Tool for Quantitative Studies, established by the Effective Public Health Practice Project, was used to evaluate the quality of included studies (Thomas et al., 2008). It assesses selection bias, study design, confounders, blinding, data collection method, and withdraws and dropouts. Each domain is rated as ‘weak,’ ‘moderate,’ or ‘strong’, which determines the overall rating for each study: studies with no weak ratings are given a global rating of strong, those with one weak rating are given a global rating of moderate, and those with two or more weak ratings are given a global rating of weak. Descriptive information was also provided with respect to intervention integrity and analysis. Two researchers (DA and SA) assessed the quality of included studies blindly and independently, and disagreements were resolved via discussion with two other researchers (RG and CM).

### Narrative synthesis

A narrative synthesis was used to summarise the findings. This is an approach that relies on the synthesis and analysis of data across various studies using texts and words (Leamy et al., 2011). There were an insufficient number of studies to conduct a meta-analysis.

## Results

### Study selection

**Figure 1.**
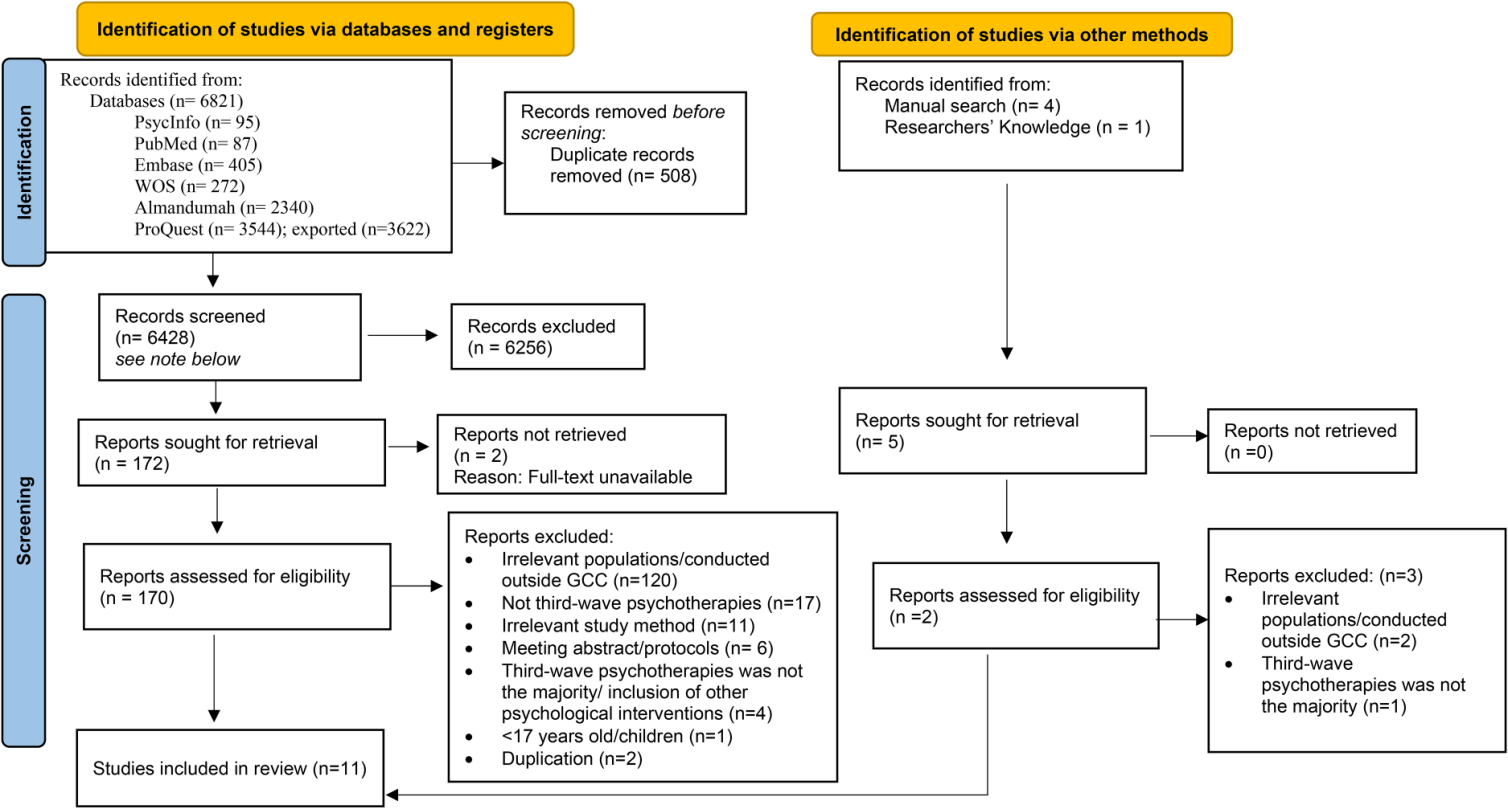
PRISMA flowchart.

*Note*. Initial records screened from database were 6,313 records. However, more records were screened (6,428) due to some of the pilot search results were being imported into the same Excel sheet.

Figure 1 presents the PRISMA flowchart. The search identified 6,821 records, with 11 studies being included in the review after screening for eligibility criteria.

### Study characteristics

Study characteristics are presented in Table 2. Five studies were CTs, five were RCTs, and one was a prospective cohort study. Seven studies were published in Arabic, and four in English. The studies were conducted in three different GCC countries (Kingdom of Saudi Arabia, State of Kuwait, and United Arab Emirates), with the majority (9/11, 82%) being conducted in the Kingdom of Saudi Arabia.

**Table 2.** Overall study characteristics.

Study	Study design	Country	Sample size (each arm)	Sample	Mean age (SD) range	Attrition rate	Outcome	Follow-up points	Assessment tools	Third-wave Intervention	Intervention duration weeks	Comparator
Abo-Zaid, 2017	CT	Saudi Arabia	15 Intervention: 8 Control: 7	Masters’s university students	NR 25–35	NR	Borderline personality symptoms Emotion regulation difficulties Mindfulness trait	Post-intervention eight-week FU	Short version Borderline Symptom List Difficulties in Emotion Regulation Scale Five Factor Mindfulness Questionnaire	DBT (only mindfulness component)	NR	No treatment
Akras, 2016	RCT	Saudi Arabia	40 Intervention: 40 Control: 40	University students	NR	NR	Psychasthenia	Post-intervention four-week FU	Psychasthenia Scale: The Minnesota Multiphasic Personality Inventory	Based on MBCT	8	No treatment
Al-Ghalib & Salim, 2018	RCT	Saudi Arabia	60 NR	Undergraduate students	NR 17–24	53%	Well-being Stress Depression Life satisfaction Mindfulness trait	Post-intervention	The Short Warwick-Edinburgh Mental Well-Being Scale The Depression Anxiety Stress Scales The Satisfaction with Life Scale The Freiburg Mindfulness Inventory	Based on MBSR	3	Analytical thinking, critical appraisal, reflection (lectures, assignments, and reading material)
Alhawatmeh et al., 2022	RCT	Kuwait	84 Intervention: 42 Control: 42	End-stage renal disease patients undergoing haemodialysis	NR	12%	Perceived stress Emotion regulation Kidney disease-related quality of life Mindfulness trait	Post-intervention	Perceived Stress Scale Emotion Regulation Questionnaire Kidney Disease-Quality of Life Questionnaire Mindful Attention Awareness Scale	Smith's version of mindfulness meditation	5	Participants were instructed to close their eyes and relax
Arnout, 2019a	CT	Saudi Arabia	26 Intervention: 13 Control: 13	Renal failure patients	40.8 (4.9) 35–48	NR	Psychological well-being	Post-intervention four-week FU	Psychological Well-being Scale for Adults with Chronic Illnesses	Based on ACT	8	No treatment
Arnout, 2019b	CT	Saudi Arabia	80 Intervention: 40 Control: 40	High-school teachers	42.5 (5) 35–52	NR	Strength of personality trait Psychological well-being	Post-intervention four-week FU	Strength of Personality Scale Teacher's Psychological Wellbeing Scale	Based on ACT In-person setting	4	Based on ACT Internet-based setting
Al-Shareef, 2020	RCT	Saudi Arabia	20 Intervention: 10 Control: 10	Older adults in care homes	66.2 4.4 (NR)	NR	Depression symptoms	Post-intervention four-week FU eight-week FU	Geriatric Depression Scale-Short term	Based on ACT	8	No treatment
Al-Sha’rawi, 2021	CT	Saudi Arabia	24 Intervention: 12 Control: 12	University students	22.2 (0.78) 22–24	NR	Psychological serenity/calm	Post-intervention eight-week FU	Psychological Serenity/Calm Scale	Based on ACT	4	No treatment
Bader, 2021	Prospective cohort	Saudi Arabia	9	Divorcees using Yosr Association Centre	NR 36–40	11%	Depression symptoms Anxiety symptoms Feeling of inferiority	Post-intervention 12-week FU	Beck Anxiety Inventory-II Feeling of Inferiority Scale	Based on ACT	16	NA
Eldabea, 2021	CT	Saudi Arabia	13 Intervention: 7 Control: 6	University students under quarantine	20.3 (0.95) NR	NR	Multi-faceted anxiety of home quarantine	Post-intervention four-week FU	Multi-Faceted Anxiety of Home Quarantine	Self-compassion	4	No treatment
Thomas et al., 2016	RCT	United Arab Emirates	24 Intervention: 12 Control: 12	College students	NR 18–27	0%	Stress reactivity Depression symptoms	Post-intervention	Daily Life Stress Scale UAE Beck Depression Inventory-II	MBSR	8	Wait list

*Notes*. ACT = Acceptance and Commitment Therapy; CT = non-randomised controlled trial; DBT = Dialectical Behavioural Therapy; FU = follow-up; MBCT = Mindfulness-Based Cognitive Therapy; MBSR = Mindfulness-Based Stress Reduction; NA = Not applicable; NR = not reported; RCT = randomised controlled trial.

Of the studies employing RCT and CT designs (10/11), seven studies compared third-wave psychotherapies to a non-active control (such as no treatment or wait list controls), two studies to an active control (such as analytical thinking, critical appraisal and reflection, and relaxation), and one study to another form of treatment (such as different delivery modes of the same intervention).

### Sample size

Sample sizes ranged from nine (Bader, 2021) to 84 (Alhawatmeh et al., 2022), with a total of 395 participants included across all studies. An a priori power analysis to determine the sample size was conducted in only two studies, with both successfully meeting the targeted sample size. Moreover, only four studies reported the total number of dropouts, and only two of these reported reasons for dropout.

### Sample characteristics

Most studies (9/11, 82%) recruited participants from non-clinical settings, such as universities and care homes. Two studies recruited participants from clinical settings (i.e., hospitals). Furthermore, six studies recruited university students, of which two involved students with self-reported mental health difficulties. Two studies recruited participants with physical health conditions. The remaining three studies recruited high-school teachers, divorcees, and older people in care homes, of which two involved participants with self-reported mental health difficulties.

Overall, participants ranged in age from 17 to 52 years old, with the overall mean age ranging from 20 to 66 years old across studies reporting these data. Participants were predominantly female in 64% (7/11) of studies. None of the included studies reported on participants’ ethnicities.

### Outcome characteristics

No physical health outcomes were identified in any study. All reported outcomes were related to mental health, which varied from psychological distress and well-being to psychological traits. All studies reported outcomes at post-intervention, and nearly all studies (8/11) reported outcomes at follow-up. Of these, seven had a four- to eight-week follow-up, and one had a 12-week follow-up. All studies referred to statistically significant changes in outcomes, with none reporting clinically important or reliable change indices of change effects.

### Intervention characteristics

The majority of studies were either based on ACT (5/11) or MBIs (4/11), with only one study examining a mindfulness component of DBT, and one study examining CBI. Nearly half (5/11) of studies did not report the mode of intervention delivery, whereas the remaining studies delivered the intervention: (a) in person (2/6); (b) using a combination of an in-person and internet-based format (2/6); (c) via an internet-based format (1/6); and (d) in one of two treatment arms (in-person vs. internet-based) (1/6). Furthermore, most studies reporting intervention delivery details were in a group format (4/6), with only two interventions being delivered individually or using a combination of group and individual delivery.

The intervention duration ranged from three weeks (Al-Ghalib & Salim, 2018) to 16 weeks (Bader, 2021). One study did not report the intervention duration (Akras, 2016). The number of sessions varied between one and three sessions per week that lasted for 30 to 180 min per session across studies reporting these data. Nearly all studies (9/11) included home practice in the intervention programme. Furthermore, four studies did not report details about the therapist(s) who conducted or delivered the intervention. The remaining interventions were mostly administered by the study authors (6/7), with only one intervention being administered by a well-being expert. Furthermore, therapists’ proficiency and degree of training were only reported in two studies (Alhawatmeh et al., 2022; Thomas et al., 2016).

### Quality assessment

The methodological quality of all but one included study was judged to be weak (see Appendix B; supplementary materials). For most studies (10/11, 91%), the type of study design was generally adequate, and the data collection methods were reliable and valid in 64% (7/11) of studies. However, selection bias was rated as weak due to self-referral or a lack of information in 73% (8/11) of studies. Additionally, 73% (8/11) did not report withdrawals and dropouts and so were rated as weak in this domain.

### Evaluation of cultural adaptation

The degree of cultural adaptation in each study based on Bernal et al.'s framework is presented in Table 3. Extracted quotes and examples are outlined in Appendix C; supplementary materials. The overall score for cultural adaptation ranged from 2 to 5 out of a possible score of 9, with a mean score of 3.4 (SD = 1) across all studies.

**Table 3.** The degree of cultural adaptation based on Bernal et al.'s framework.

Study	Language	Person	Metaphor	Content		Goal	Concept	Method	Context	Total 0–9
				Assessment tools	Intervention content					
Abo-Zaid, 2017	1	0	0	1	0	0	0	0	0	2
Akras, 2016	1	0	0	1	0	1	0	1	0	4
Al-Ghalib & Salim, 2018	1	0	1	0	1	0	0	1	1	5
Alhawatmeh et al., 2022	1	0	0	1	0	0	0	1	1	4
Arnout, 2019a	1	0	0	1	0	1	0	0	1	4
Arnout, 2019b	1	0	0	1	0	1	0	1	0	4
Al-Shareef, 2020	1	0	0	1	0	0	0	0	0	2
Al-Sha’rawi, 2021	1	0	0	1	0	0	0	0	0	2
Bader, 2021	1	0	0	1	0	1	0	1	1	5
Eldabea, 2021	1	0	0	1	0	0	0	1	0	3
Thomas et al., 2016	0	0	0	1	0	0	0	1	1	3

*Note*. Details about the cultural adaptations in each study are presented in Appendix C; supplementary materials.

The majority of studies (10/11, 91%) were assumed to have culturally adapted the intervention with respect to language since the included studies were conducted in the GCC and therefore were assumed to have been delivered in Arabic. However, only two studies explicitly reported the language of the intervention: one in Arabic (Alhawatmeh et al., 2022) and one in English (Thomas et al., 2016).

With respect to adaptations to assessment tools and intervention content (e.g., by incorporating knowledge of the local culture), most studies (10/11, 91%) adapted assessment tools by using an Arabic version of the scale (Alhawatmeh et al., 2022; Bader, 2021; Thomas et al., 2016), conducting an Arabic translation of the scale (Abo-Zaid, 2017), explicitly stating that the scales had been used before with similar populations and/or cultures (Akras, 2016; Alhawatmeh et al., 2022; Arnout, 2019b; Bader, 2021), or developing new scales to ensure their suitability for the target population and/or culture (Al-Sha’rawi, 2021; Arnout, 2019a; Arnout, 2019b; Eldabea, 2021; Thomas et al., 2016). The method of assessment tool adaptation was unclear in one study (Al-Shareef, 2020). In contrast, only one study explicitly adapted intervention content by modifying it with respect to Islamic spirituality (Al-Ghalib & Salim, 2018). For example, the inclusion of spiritual awareness involved *Taqwa* (being conscious of God's presence) and the practice of *Dhikr* (chanting).

Method adaptation, which refers to the practical aspects of delivering interventions informed by the knowledge of the population and/or local culture, was utilised in 64% (7/11) of studies. For example, Alhawatmeh et al. (2022) conducted brief sessions based on previous recommendations that suggested a brief intervention programme might lead to greater benefit for patients undergoing haemodialysis.

Several studies (5/11, 45%) utilised context adaptation, which refers to a broader contextual discussion that is relevant to the local culture but not necessarily incorporated into the intervention plan. Examples include a discussion of mindfulness in relation to Islamic societies and the need for such programmes to meet the new political vision of Saudi Arabia (Al-Ghalib & Salim, 2018), and participants highlighting that their current problems were a result of social stress and society's negative views (Bader, 2021). Likewise, several studies (4/11, 36%) also utilised goal adaptation, which emphasises the discussion and/or consistency of intervention goals with participants’ values. For example, Arnout (2019a) incorporated a discussion with participants in which they contributed to goal setting, and Bader (2021) ensured that participants’ goals and expectations met the intervention's main goals.

Only one study (9%) utilised metaphor adaptation (i.e., sayings, stories, and symbols adapted to the local culture), whereby videos and reading materials were adapted to recognise and empower Islamic spirituality (Al-Ghalib & Salim, 2018). There was insufficient information about the characteristics of therapists who conducted the interventions, including their ethnicities, in all studies; therefore, it is unclear whether any of the interventions utilised person adaptation. Finally, none of the interventions utilised concept adaptation, which emphasises whether the conceptualisation of the intervention or the presentation of participants’ distress was adapted to the local culture.

### Effectiveness of third-wave psychotherapies

#### Psychological well-being

The effectiveness of third-wave psychotherapies on psychological well-being (i.e., well-being, life satisfaction, quality of life, and psychological serenity/calm) was examined in five studies (see Table 4). Three studies examined ACT (Al-Sha’rawi, 2021; Arnout, 2019a; Arnout, 2019b), and all reported a statistically significant increase in psychological well-being among participants who received ACT compared to a non-active control condition or in-person intervention arm at post-intervention. Maintenance of benefits of ACT at four- to eight-week follow-up was also reported in these studies. In contrast, findings were mixed for MBIs: one study reported a statistically significant increase in psychological well-being among participants who received MBI compared to an active control condition at post-intervention (Alhawatmeh et al., 2022), while another did not (Al-Ghalib & Salim, 2018).

**Table 4.** Results of all included outcomes.

Study	Statistical analysis	Total analysed sample	Outcome	Main findings	Additional information
**Abo-Zaid, 2017**	Mann-Whitney U Test Wilcoxon-Z Test	NR	Borderline personality symptoms Emotion regulation difficulties Mindfulness trait	Statistically significant: (a) decrease in borderline personality symptoms and emotion regulation difficulties, and (b) increase in mindfulness trait among Master’s university students who received mindfulness component of DBT compared to no treatment control at post-intervention. Statistically significant: (a) decrease in borderline personality symptoms and emotion regulation difficulties, and (b) increase in mindfulness trait between pre-intervention and post-intervention scores among Master’s university students who received mindfulness component of DBT. No statistically significant differences in borderline personality symptoms, emotion regulation difficulties, and mindfulness trait between post-intervention and eight-week FU scores among Master’s university students who received mindfulness component of DBT; maintenance of intervention benefits.	Between-group differences were not reported at eight-week FU.
**Akras, 2016**	Paired-sample T-test	40	Psychasthenia	Statistically significant decrease in psychasthenia among university students who received intervention based on MBCT compared to no treatment control at post-intervention and at four-week FU. No statistically significant differences in psychasthenia between post-intervention and four-week FU scores among university students who received intervention based on MBCT; maintenance of intervention benefits.	
**Al-Ghalib and Salim, 2018**	Paired-sample T-test	26	Well-being Stress Depression Life-satisfaction Mindfulness trait	No statistically significant differences in well-being, stress, depression, life-satisfaction, or mindfulness trait among undergraduate university students who received intervention based on MBSR compared to active control at post-intervention. Statistically significant increase in life satisfaction between pre-intervention and post-intervention scores among undergraduate university students who received intervention based on MBSR.	Results of pre–post differences for other outcomes were not reported. According to the study: A comparison of pre–post mean scores showed that mindfulness trait and well-being levels marginally increased, while anxiety, depression, and stress levels slightly decreased for the intervention group.
**Alhawatmeh et al., 2022**	ANOVA	74	Perceived stress Emotion regulation Kidney disease-related quality of life Mindfulness trait	Statistically significant: (a) increase in emotion regulation, kidney disease-related quality of life, and mindfulness trait, and (b) decrease in perceived stress among end-stage renal disease patients undergoing haemodialysis who received Smith's version of mindfulness meditation compared to active control at post-intervention. Statistically significant increase in emotion regulation, kidney disease-related quality of life, and mindfulness trait between pre-intervention and post-intervention scores (effect of time) for patients who received Smith's version of mindfulness meditation.	Inconsistent reporting of the significant result of perceived stress pre–post scores (effect of time) in which the result was (F = 4.60; p = 0.03); however, it was reported as non-significant in the text.
**Arnout, 2019a**	Paired-sample T-test	NR	Psychological well-being	Statistically significant increase in psychological well-being among renal failure patients who received intervention based on ACT compared to no treatment control at post-intervention. Statistically significant increase in psychological well-being between pre-intervention and post-intervention scores among patients who received intervention based on ACT. No statistically significant differences in psychological well-being between post-intervention and FU scores among patients who received intervention based on ACT; maintenance of intervention benefits.	Between-group differences were not reported at FUs. Unclear results of FU whether were at four-week or eight-week FU
**Arnout, 2019b**	Paired-sample T-test	80	Strength of personality trait Psychological well-being	Statistically significant increase in strength of personality trait and psychological well-being among high-school teachers who received internet-based ACT compared to those who received in-person of the same ACT at post-intervention and four-week FU. Statistically significant increase in strength of personality trait and psychological well-being between pre-intervention and post-intervention scores for both in-person and internet-based modes among high-school teachers who received intervention based on ACT. No statistically significant differences in strength of personality trait and psychological well-being between post-intervention and four-week FU scores for both in-person and internet-based modes; maintenance of interventions benefits.	
**Al-Shareef, 2020**	Mann-Whitney U Test Wilcoxon-Z Test	20	Depression symptoms	Statistically significant decrease in depression symptoms among older adults in care homes who received intervention based on ACT compared to no treatment control at post-intervention. Statistically significant decrease in depression symptoms between pre-intervention and post-intervention scores among older adults who received intervention based on ACT. Statistically significant decrease in depression symptoms between post-intervention to four-week FU to eight-week FU scores among older adults who received intervention based on ACT; continuation of intervention benefits.	Between-group differences were not reported at FUs.
**Al-Sha’rawi, 2021**	Mann-Whitney U Test Wilcoxon-Z Test	24	Psychological serenity/calm	Statistically significant increase in psychological serenity/calm among university students who received intervention based on ACT compared to no treatment control at post-intervention. Statistically significant increase in psychological serenity/calm between pre-intervention and post-intervention scores among university students who received intervention based on ACT. No statistically significant differences in psychological serenity/calm between post-intervention and eight-week FU scores among university students who received intervention based on ACT; maintenance of intervention benefits.	Between-group differences were not reported at FU. Study also reported between- and within-group differences of each psychological serenity/calm scale domain.
**Bader, 2021**	Wilcoxon-Z Test	8	Depression symptoms Anxiety symptoms Feeling of inferiority	Statistically significant decrease in depression symptoms, anxiety symptoms, and feeling of inferiority between pre-intervention and post-intervention scores among divorcees who received intervention based on ACT. No statistically significant differences in depression symptoms, anxiety symptoms, and feeling of inferiority between post-intervention and 12-week FU scores among divorcees who received intervention based on ACT; maintenance of intervention benefits.	
**Eldabea, 2021**	Mann-Whitney U Test Wilcoxon-Z Test	13	Multi-faceted anxiety of home quarantine	Statistically significant decrease in anxiety among university students under quarantining who received self-compassion compared to no treatment control at post-intervention. Statistically significant decrease in anxiety between pre-intervention and post-intervention scores among university students under quarantining who received self-compassion. No statistically significant differences in anxiety between post-intervention and four-week FU scores among university students under quarantining who received mindful self-compassion; maintenance of intervention benefits.	Between-group differences were not reported at FU. Study also reported between- and within-group differences of each multi-faceted anxiety of home quarantine scale domain.
**Thomas et al., 2016**	ANCOVA	24	Stress reactivity Depression symptoms	Statistically significant decrease in stress reactivity and depression symptoms among college students who received MBSR compared to wait list at post-intervention, after adjusting for pre-intervention scores.	

*Notes*. ACT = Acceptance and Commitment Therapy; ANCOVA = analysis of covariance; ANOVA = analysis of variance; DBT = Dialectical Behavioural Therapy; FU = follow-up; MBCT = Mindfulness-Based Cognitive Therapy; MBSR = Mindfulness-Based Stress Reduction; NR = not reported.

#### Psychological distress

The effectiveness of third-wave psychotherapies on psychological distress (i.e., depression, stress, anxiety, emotional regulation/difficulties, psychasthenia, borderline personality symptoms, and feelings of inferiority) was reported in eight studies, six of which reported on depression, stress, and anxiety (see Table 4). Findings were consistent across these six studies, with the majority (5/6) reporting either a statistically significant decrease in depression, stress, and/or anxiety symptoms among participants who received third-wave psychotherapies compared to non-active or active control conditions at post-intervention, or a pre–post reduction in symptoms. Two of these studies examined MBIs (Alhawatmeh et al., 2022; Thomas et al., 2016), two examined ACT (Al-Shareef, 2020; Bader, 2021), and one examined CBI (Eldabea, 2021). Of these studies, three reported either maintenance or continuous benefits of ACT or CBI at four- to 12-week follow-up. Only one study reported non-significant differences in depression and stress among participants who received MBI compared to an active control condition at post-intervention (Al-Ghalib & Salim, 2018).

Two studies that examined MBIs reported a statistically significant improvement in emotional regulation compared to non-active or active control conditions at post-intervention, with only one reporting maintenance of benefits at eight-week follow-up (Abo-Zaid, 2017; Alhawatmeh et al., 2022). Psychasthenia, borderline personality symptoms, and feelings of inferiority were only reported in one study each and so are not further described here (Abo-Zaid, 2017; Akras, 2016; Bader, 2021).

#### Psychological traits

The effectiveness of third-wave psychotherapies on psychological traits (i.e., mindfulness and personality traits) was reported in four studies (see Table 4). Findings were mixed with respect to mindfulness traits: two out of three studies that examined MBIs reported statistically significant improvements in mindfulness traits compared to non-active or active control conditions at post-intervention, with one reporting maintenance of benefits at eight-week follow-up (Abo-Zaid, 2017; Alhawatmeh et al., 2022). In contrast, one study reported no statistically significant improvements in mindfulness traits among participants who received MBI compared to an active control condition at post-intervention (Al-Ghalib & Salim, 2018). Only one study examined a personality trait of strength (defined by the study as an individual's capability to be strong in making decisions, live with enthusiasm, achieve goals, etc.) (Arnout, 2019b), and so this is not further described here.

## Discussion

This systematic review critically evaluated cultural adaptations to third-wave psychotherapies and explored the effectiveness of these interventions on physical and mental health outcomes in GCC populations. Third-wave psychotherapies included ACT, MBIs, DBT, and CBI.

The overall degree of cultural adaptation based on Bernal et al.'s framework was relatively inadequate (mean = 3.4, SD = 1). The most frequently used adaptation was assumed to be language given that the included studies were conducted in the GCC and therefore were assumed to have been delivered in Arabic. However, as only two studies explicitly reported the language of the intervention (one in Arabic and one in English), this assumption should be treated with caution. It is important to culturally adapt the language of any intervention as it may lead to greater therapeutic efficacy; for example, it might help clients to better express their emotions (Bernal & Sáez-Santiago, 2006). Future studies should clearly report on the language of intervention delivery so that the degree of adaptation can be evaluated with greater certainty.

Likewise, assessment tool adaptations were used by most studies, whereby studies explicitly stated that scales were previously validated in similar populations and/or cultures. Such cultural adaptations mean that assessment tools were locally appropriate and valid, which may have improved their accuracy to detect symptoms compared to tools that were not adapted (Ali et al., 2016; Atilola, 2015). Some studies, however, reported translating assessment tools into Arabic, but others have argued that direct and simple translation does not guarantee cultural appropriateness (Cha et al., 2007). Some studies also reported creating new scales to suit the targeted populations/culture. However, this might lead to issues concerning the validity and reliability of the scales, particularly if they were not previously piloted, as both validity and reliability are needed to ensure data replicability, accuracy, and integrity (Mohajan, 2017). Future studies should ensure that assessment tools are valid within the local culture and demonstrate good psychometric properties within that culture.

Several studies used goal, method, and context adaptations; for example, agreement of intervention goals in accordance with participants’ values, use of practical procedures that were congruent with populations/cultures, and broader contextual discussions that were relevant to populations/cultures. Failing to adapt these domains might lead to incompatibility between intervention procedures and a participant’s culture or discrepancies in intervention goals between the therapist and client. Both of these could affect the therapeutic relationship and therapist credibility (Bernal et al., 1995), which could in turn reduce the effectiveness of any intervention. Consequently, future studies should put greater emphasis on cultural adaptations that could affect intervention efficacy.

In contrast to other adaptations, only one study explicitly adapted metaphor and intervention content, and no studies applied person or concept adaptations. It is difficult to know whether these adaptations were simply not conducted or not explicitly reported. The fact that studies were conducted in the GCC might support either interpretation. It has been argued that interventions conducted in a homogenous culture, which is the case for GCC populations who share similar systems and cultures, might be less likely to report details such as therapists’ ethnicity than interventions conducted in a multicultural society (Castellanos et al., 2020). Explicit reporting of intervention elements and methods is crucial in healthcare research, especially when interventions are developed in a different culture, as failing to do this makes it difficult to estimate the degree to which cultural adaptations are implemented. Therefore, future studies should explicitly and comprehensively report on intervention adaptations that have been implemented. If metaphor, intervention content, person, and concept adaptations were simply not conducted rather than not being explicitly reported, this suggests that future studies should ensure they incorporate these cultural adaptations to enhance the cultural sensitivity of any intervention and potentially increase its efficacy (Bernal et al., 1995).

Previous reviews of cultural adaptations to psychological interventions based on Bernal et al.'s framework (Castellanos et al., 2020; Shehadeh et al., 2016) have reported a similar pattern of findings with respect to implemented cultural adaptations. Similar to this review, language has been reported to be the most frequently used adaptation, with few studies reporting adaptations to the method, context, metaphor, and content across reviews. However, the evaluations of concept, person, and goal adaptations are inconsistent across reviews. For example, Castellanos et al.'s review found that 16 studies included person adaptation while none of the studies in this review did, and only one study incorporated goal adaptation in the previous reviews (Castellanos et al., 2020; Shehadeh et al., 2016), whereas it was adapted in four studies in this review. This inconsistency might be due to: (a) heterogeneity in definitions of Bernal et al.'s dimensions, as they are not distinct but overlap; (b) differences in the degree of explicit reporting of cultural adaptations across studies; or (c) differences in the adaptation of psychological interventions across populations and cultures.

To the authors’ knowledge, no previous reviews have systematically evaluated cultural adaptations to third-wave psychotherapies in the GCC. A similar review of cultural adaptations to positive psychology interventions in Arab countries was conducted recently (Basurrah et al., 2022b). Although these authors found that most of these interventions (91%) were culturally adapted, there are a number of reasons why the results of this previous review cannot be extrapolated to the current review of third-wave psychotherapies in the GCC. First, only 24% of studies in Basurrah et al.'s review were conducted in the GCC, so findings are likely to be less relevant to GCC culture. Second, Basurrah et al. reported limited details with respect to cultural adaptation domains and the cultural evaluation method used, so there is insufficient information to critically evaluate the degree of cultural adaptation employed in their included studies. Third, only MBIs and CBIs were included in Basurrah et al.'s review as representatives of third-wave psychotherapies, which limits the generalisability of findings to broader third-wave psychotherapies. Clearly, further reviews are needed to explore the cultural appropriateness and effectiveness of psychological interventions in the GCC.

Turning to the effectiveness of culturally adapted third-wave psychotherapies for physical and mental health outcomes in GCC populations, the majority of studies (10/11, 91%) reported statistically significant findings in relation to mental health outcomes in comparison to control or comparator conditions. These outcomes included psychological well-being, distress, and psychological traits. Furthermore, more than half of the studies (8/11, 73%) reported maintenance of gains at follow-up. This suggests that the effects of these interventions are maintained beyond post-intervention. However, no physical health outcomes were identified, which suggests future studies should explore physical health-related outcomes (e.g., chronic pain).

Furthermore, several issues need to be addressed with respect to the adaptation of psychological interventions in GCC. First, it is crucial to consider the attitudes of GCC populations towards mental health in general and their perceptions of receiving psychological interventions for mental health difficulties. Understanding societal perspectives might help in culturally adapting psychological interventions, thus enhancing their acceptability. Elyamani et al. (2021) found a lack of knowledge about mental health among the GCC public and, surprisingly, among healthcare providers, with practitioners reportedly having limited recognition of mental health conditions (e.g., post-traumatic stress disorder). They also reported high levels of stigma and shame associated with mental health difficulties, as well as negative attitudes towards people experiencing such issues. Both stigma and the lack of awareness contribute to the tendency among GCC people to seek help late and to disregard mental health interventions, which may worsen mental health conditions (Kronfol et al., 2018). Furthermore, a lack of awareness and stigma were also reported among Arab populations (Gearing et al., 2013; Maalouf et al., 2019). To address these issues, several suggestions have been recommended for Arab culture (Gearing et al., 2013); for example, aligning mental health interventions with healthcare systems, which are already widely accepted, collaborating closely with other health practitioners, and involving community leaders (e.g., older people) to promote these interventions. Consequently, it is important to ensure that people in the GCC have access to mental health services and essential knowledge of mental health without fear of negative judgement from others or associated stigma (Alzahrani, 2020). Future studies could further explore the adoption of these strategies in the GCC.

Second, the experiences of mental health service users in the GCC have highlighted various cultural beliefs and sociocultural factors that need to be considered when delivering psychological interventions in the GCC (Hickey et al., 2016). For example, there is a preference for seeking an initial consultation from traditional healing practices, such as faith healers, before seeking professional help for mental health needs. In fact, mental health service users perceived traditional healing practices to be helpful in their recovery of mental health difficulties. This preference has also been observed among Arabs (Gearing et al., 2013). Consequently, incorporating such cultural beliefs and sociocultural factors into the therapeutic process may contribute to more comprehensive mental health services in the GCC (Hickey et al., 2016). One example of integration of cultural beliefs involves including traditional healing practices within mental health services to enhance their acceptability and reduce stigma (Gearing et al., 2013; Hickey et al., 2016). Therefore, a thorough understanding of GCC cultural beliefs including identification of any social obstacles or facilitators of engagement in psychological interventions, and exploration of the experiences of relevant stakeholders (e.g., mental health service users and practitioners) are vital when implementing and adapting psychological interventions. Future research, particularly qualitative studies, is needed to address these aspects.

Third, third-wave psychotherapies might be considered self-directed approaches as they emphasise the person's relationship to their thoughts, emotions, values, and experiences. The self-directed perspective is often associated with individualistic cultures that strengthen personal uniqueness, as observed in Western countries (Basurrah et al., 2022a; Markus & Kitayama, 1991). While the GCC, as part of the Arab countries, may exhibit some degree of individualism, they are predominantly influenced by collectivist culture which is shaped by social norms and connections (Basurrah et al., 2022a). In collectivist cultures, people may construct their sense of self through different means, including their relationships with others rather than solely focusing on the inner aspects, as is the case in individualist cultures (Markus & Kitayama, 1991). Therefore, it is crucial to consider the influence of individualism-collectivism frameworks when adapting and evaluating any psychological interventions to enhance their acceptability among the GCC population. Future research should explore this further.

Finally, several critical issues have been identified in mental health policy and politics in the GCC (Kronfol et al., 2018). One issue is that mental health services in the GCC are predominantly provided via hospital-based facilities rather than community-based or primary healthcare sittings. This contradicts previous recommendations for Arab culture (e.g., alignment with healthcare systems, involvement of community members), as discussed earlier (Gearing et al., 2013). Another issue is that although a few policy initiatives have been implemented to address cultural adaptations (e.g., adapting assessment tools), the GCC mostly relies on mental health guidelines established in other cultures (e.g., United States, United Kingdom), resulting in a lack of culturally specific mental health guidelines for the GCC (Kronfol et al., 2018). Consequently, policymakers and relevant stakeholders should further invest in the cultural adaptation of mental health services and interventions, aligning with the GCC's future national growth visions (e.g., the Saudi healthcare 2030 vision).

### Strengths and limitations

To the authors’ knowledge, this pre-registered systematic review is the first to critically evaluate cultural adaptations to third-wave psychotherapies and to explore the effectiveness of these interventions in GCC populations. However, this review has several limitations. First, the evaluation of cultural adaptation was dependent on what was explicitly reported in studies. This means that some cultural adaptations might not have been reported due to included studies being conducted in the GCC (as noted earlier). Therefore, some uncertainty remains with respect to the degree of cultural adaptation in included studies.

Second, this review used Bernal et al.'s adaptation framework, following previous reviews (Castellanos et al., 2020; Shehadeh et al., 2016). However, others have argued that this framework is difficult to implement in real-world settings and that its eight dimensions are not distinct but overlap. For example, it has been argued that it is challenging to differentiate between language and metaphor adaptation, as both are closely linked to one another (Chu & Leino, 2017; Heim & Kohrt, 2019). Hence, this review's findings are subject to the limitations of Bernal et al.'s adaptation framework.

Third, although the majority of electronic databases were screened by two independent raters, one electronic database (Almandumah) was screened by only one rater, which may have introduced a degree of selection bias. Furthermore, although steps were taken to minimise the chances of missing relevant studies (e.g., by double checking searches), some studies might have been missed, which may have altered the conclusions drawn here.

Fourth, the number of included studies was relatively small (K = 11), and the methodological quality was judged to be weak in most of the studies (10/11), leading to possible concerns about the credibility of studies’ findings (Furuya-Kanamori et al., 2021). Furthermore, a priori power calculations were only performed by two studies, which means that it is unclear whether the remaining studies had a sufficient sample size to examine the effectiveness of third-wave psychotherapies on outcomes. Performing a priori power calculations is important for determining the sample size needed to detect meaningful effects (Kyonka, 2019). Therefore, the findings of this systematic review should be interpreted with some degree of caution.

Fifth, none of the studies compared the effectiveness of culturally adapted third-wave psychotherapies to non-culturally adapted third-wave psychotherapies. Also, none examined the effect of culturally adapted vs. non-culturally adapted third-wave psychotherapies on attrition rates. Therefore, the extent to which culturally adapting third-wave psychotherapies may impact on intervention efficacy or attrition rate remains unclear. Clearly, further research is needed to examine this. Such information could advance understanding of the importance of culturally adapted third-wave psychotherapies and, more broadly, of psychological interventions. Furthermore, although more than half of the controlled trials included a follow-up point, only two studies compared the intervention and control conditions at follow-up. Therefore, maintenance of gains in third-wave interventions vs. control conditions remains unclear beyond post-intervention.

Sixth, the generalisability of this review's findings is limited by the fact that most participants were female and most studies were conducted in the Kingdom of Saudi Arabia. This means that the results of the review may not generalise to broader populations. Furthermore, no studies reported physical health outcomes. Therefore, this review was unable to evaluate the effect of culturally adapted third-wave psychotherapies on physical health.

Seventh, third-wave psychotherapies, as a group, remain highly diverse, with each type potentially having different impacts on outcomes due to variations in therapeutic components and mechanisms of change. Although the effectiveness of third-wave psychotherapies in this review was evaluated separately based on their types, further examination of specific types within third-wave psychotherapy, with a greater number of future studies for each type, is needed. This may have influenced the conclusion drawn here.

Finally, all studies referred to statistically significant changes in outcomes, with none reporting clinically important or reliable change indices of effects. It is important to determine whether functional health outcomes are not only statistically significant but also clinically meaningful (Ferguson et al., 2002). Therefore, recommendations cannot be made with respect to clinical practice at this time.

### Future implications

Future research with more rigorous methodologies is needed to examine the cultural appropriateness and effectiveness of third-wave psychotherapies in GCC populations given that third-wave psychotherapies have been developed and evaluated in different cultures (e.g., Western cultures). In order to facilitate this, future research in GCC countries should explicitly report any cultural considerations implemented into intervention plans. Doing so might facilitate the exploration of which adaptation modifications enhance the acceptability and effectiveness of psychological interventions for different cultural contexts (Heim & Kohrt, 2019). In addition to recommendations noted above, future research should also investigate the effectiveness of third-wave psychotherapies in comparison to controls at follow-up in GCC populations, as well as recruiting more diverse populations in order that results can be generalised to broader populations.

## Conclusion

This review aimed to critically evaluate cultural adaptations to third-wave psychotherapies and explore the effectiveness of these interventions for mental and physical health outcomes in GCC populations. Culturally adapted third-wave psychotherapies were associated with improvements in numerous mental health outcomes for GCC individuals in most of the studies (10/11). Although these findings are promising, they should be interpreted with caution due to the small number of studies conducted in the GCC, cultural adaptation evaluation relying on explicit reporting in studies, the weak methodological quality of the included studies, and participants’ characteristics. Therefore, it is not possible to draw strong conclusions with respect to the cultural appropriateness and effectiveness of third-wave psychotherapies for GCC populations at this time. Future rigorous research is needed in the GCC, with more comprehensive reporting of any cultural considerations implemented into third-wave psychotherapies.

## Registration and protocol

This review follows the preferred reporting items for systematic reviews and meta-analyses (PRISMA) (Page et al., 2021). See Appendix D; supplementary materials for the PRISMA checklist. The protocol for this review was pre-registered with the Open Science Framework (10.17605/OSF.IO/SMYJW). Only one amendment was made to the protocol (see Appendix E; supplementary materials).

## Supplemental Material

sj-docx-1-tps-10.1177_13634615241227691 - Supplemental material for Cultural adaptations of third-wave psychotherapies in Gulf Cooperation Council countries: A systematic review Supplemental material, sj-docx-1-tps-10.1177_13634615241227691 for Cultural adaptations of third-wave psychotherapies in Gulf Cooperation Council countries: A systematic review by Duaa H. Alrashdi, Aisha H. Alyafei, Samar A. Alanazi, Carly Meyer, and Rebecca L. Gould in Transcultural Psychiatry
