# Supplementary material for: Cultural adaptations of third-wave psychotherapies in Gulf Cooperation Council countries: A systematic review
Source: Transcult Psychiatry. 2024 Feb 8;61(2):209–28. doi: 10.1177/13634615241227691 (PMC10943625; doi:10.1177/13634615241227691)
Supplement: sj-docx-1-tps-10.1177_13634615241227691 - Supplemental material for Cultural adaptations of third-wave psychotherapies in Gulf Cooperation Council countries: A systematic review [file sj-docx-1-tps-10.1177_13634615241227691.docx]

**Cultural Adaptations of Third-Wave Psychotherapies in Gulf Cooperation Council countries: A Systematic Review.**

Table of Contents

[Appendix A: Search terms and strategy 2](#_Toc124785889)

[Appendix B: The Quality Assessment Tool for Quantitative Studies. 6](#_Toc124785890)

[Appendix C: Rating, extracted quotes, and examples of adaptation dimensions for each study 8](#_Toc124785891)

[Appendix D: PRISMA checklist 12](#_Toc124785892)

[Appendix E: Protocol amendments 15](#_Toc124785893)

# Appendix A: Search terms and strategy

Four electronic databases (PubMed, Web of Science, PsycInfo, and Embase), one electronic Arabic database (Almandumah, accessed via the Saudi Digital Library), and grey literature (via ProQuest) were searched separately from the date of conception to 16–23 May 2022. English and Arabic search terms were developed based on key concepts related to third-wave psychotherapies, the GCC, Arabs, and Islam.

Embase

1 exp mindfulness

2 exp meditation/ or exp mindfulness meditation

3 mindful*.ti,ab.

4 meditat*.ti,ab.

5 MBCT.ti,ab.

6 MBSR.ti,ab.

7 acceptance-based.ti,ab.

8 ("acceptance and commitment therapy" or "acceptance commitment therapy" or "acceptance commitment training" or "acceptance and commitment training").mp.

9 'compassionate mind training'.mp.

10 ("compassion-focused therapy" or "compassion focused therapy" or CFT).mp.

11 DBT.ti,ab.

12 ("dialectical behavior therapy" or "dialectical behavioral therapy" or "dialectical behaviour therapy" or "dialectical behavioural therapy").mp.

13 1 or 2 or 3 or 4 or 5 or 6 or 7 or 8 or 9 or 10 or 11 or 12

14 exp Islam/

15 exp Qatar/

16 Qatar*.mp.

17 exp Kuwait/

18 Kuwait*.mp.

19 exp Saudi Arabia/

20 Saudi*.mp.

21 exp Bahrain/

22 Bahrain*.mp.

23 exp Oman/ or exp "Gulf of Oman"/

24 Oman*.mp.

25 exp United Arab Emirates/

26 "United Arab Emirates".mp.

27 Emirat*.mp.

28 UAE.mp.

29 exp Arab world/ or exp Arab/

30 Arab*.mp.

31 exp Middle East/

32 "Middle East".mp.

33 ("Gulf country" or "Gulf countries").mp.

34 14 or 15 or 16 or 17 or 18 or 19 or 20 or 21 or 22 or 23 or 24 or 25 or 26 or 27 or 28 or 29 or 30 or 31 or 32 or 33

35 13 and 34

PsycInfo

1 exp Mindfulness-Based Interventions/ or exp Mindfulness/

2 exp Meditation/

3 mindful*.ti,ab.

4 meditat*.ti,ab.

5 MBCT.ti,ab.

6 MBSR.ti,ab.

7 acceptance-based.ti,ab.

8 ("acceptance and commitment therapy" or "acceptance commitment therapy" or "acceptance commitment training" or "acceptance and commitment training").mp.

9 "compassionate mind training".mp.

10 ("compassion-focused therapy" or "compassion focused therapy" or CFT).mp.

11 DBT.ti,ab.

12 ("dialectical behavior therapy" or "dialectical behavioral therapy" or "dialectical behaviour therapy" or "dialectical behavioural therapy").mp.

13 1 or 2 or 3 or 4 or 5 or 6 or 7 or 8 or 9 or 10 or 11 or 12

14 exp Islam/

15 Qatar*.mp.

16 Kuwait*.mp.

17 Saudi*.mp.

18 Bahrain*.mp.

19 Oman*.mp.

20 "United Arab Emirates".mp.

21 Emirat*.mp.

22 UAE.mp.

23 exp Arabs/

24 Arab*.mp.

25 "Middle East".mp.

26 ("Gulf country" or "Gulf countries").mp.

27 14 or 15 or 16 or 17 or 18 or 19 or 20 or 21 or 22 or 23 or 24 or 25 or 26

28 13 and 27

Web of Science

mindful* or meditat* or MBCT or MBSR or 'acceptance-based' or 'acceptance and commitment therapy' or 'acceptance commitment therapy' or 'acceptance commitment training' or 'acceptance and commitment training' or 'compassionate mind training' or 'compassion-focused therapy' or 'compassion focused therapy' or CFT or DBT or 'dialectical behavior therapy' or 'dialectical behavioral therapy' or 'dialectical behaviour therapy' or 'dialectical behavioural therapy'

and

Qatar* or Kuwait* or Saudi* or Bahrain* or Oman* or 'United Arab Emirates' or Emirat* or UAE or Arab* or 'Gulf country' or 'Gulf countries' or 'Middle East' or Islam

PubMed

mindful* or meditat* or MBCT or MBSR or "acceptance-based" or "acceptance and commitment therapy" or "acceptance commitment therapy" or "acceptance and commitment training" or "compassionate mind training" or "compassion-focused therapy" or "compassion focused therapy" or CFT or DBT or "dialectical behavior therapy" or "dialectical behavioral therapy" or "dialectical behaviour therapy" or "dialectical behavioural therapy"

and

Qatar* or Kuwait* or Saudi* or Bahrain* or Oman* or "United Arab Emirates" or Emirat* or UAE or Arab* or "Gulf country" or "Gulf countries" or "Middle East" or Islam

ProQuest

[ab(mindfulness OR meditation OR mindfulness-based interventions OR 'acceptance AND commitment therapy' OR 'compassion therapy' OR dialectical behavioral therapy') AND (Arab OR Islam)](https://www.proquest.com/myresearch/savedsearches.checkdbssearchlink:rerunsearch/2159488/SavedSearches?t:ac=SavedSearches)

المنظومة (via Saudi Digital Library)  Almandumah

جميع الحقول: اليقظة الذهنية أو جميع الحقول: "العلاج السلوكي الجدلي" أو جميع الحقول: "العلاج القائم على اليقظة الذهنية" أو جميع الحقول: (("العلاج بالتقبل والالتزام") أو (العلاج بالقبول والالتزام")) أو جميع الحقول: التأمل أو جميع الحقول: )"العلاج القائم على التعاطف").

# Appendix B: The Quality Assessment Tool for Quantitative Studies.

| **Study** | **Selection bias** | **Study design** | **Confounders** | **Blinding** | **Data collection method** | **Withdrawals and dropout** | **Intervention integrity** | **Analysis** | **Overall rating** |
| --- | --- | --- | --- | --- | --- | --- | --- | --- | --- |
| **Abo-Zaid, 2017** | Moderate | Strong | Strong | Weak | Strong | Weak | (Q1) cannot tell  (Q2) cannot tell  (Q3) Yes | (Q1) Individual  (Q2) Individual  (Q3) Yes  (Q4) cannot tell | Weak |
| **Akras, 2016** | Weak | Strong | Strong | Moderate | Strong | Weak | (Q1) cannot tell  (Q2) cannot tell  (Q3) Yes | (Q1) Individual  (Q2) Individual  (Q3) Yes  (Q4) Yes | Weak |
| **Al-Ghalib & Salim, 2018** | Weak | Strong | Weak | Weak | Strong | Weak | (Q1) cannot tell  (Q2) cannot tell  (Q3) Yes | (Q1) Individual  (Q2) Individual  (Q3) Yes  (Q4) No | Weak |
| **Alhawatmeh et al., 2022** | Strong | Strong | Weak | Moderate | Strong | Strong | (Q1) cannot tell  (Q2) Yes  (Q3) Yes | (Q1) Individual  (Q2) Individual  (Q3) Yes  (Q4) No | Moderate |
| **Arnout, 2019a** | Weak | Strong | Strong | Moderate | Weak | Weak | (Q1) cannot tell  (Q2) cannot tell  (Q3) Yes | (Q1) Individual  (Q2) Individual  (Q3) Yes  (Q4) cannot tell | Weak |
| **Arnout, 2019b** | Weak | Strong | Strong | Weak | Moderate | Weak | (Q1) cannot tell  (Q2) cannot tell  (Q3) Yes | (Q1) Individual  (Q2) Individual  (Q3) Yes  (Q4) Yes | Weak |
| **Al-Shareef, 2020** | Weak | Strong | Strong | Moderate | Strong | Weak | (Q1) Less than 60%  (Q2) cannot tell  (Q3) Yes | (Q1) Individual  (Q2) Individual  (Q3) Yes  (Q4) Yes | Weak |
| **Al-Sha’rawi, 2021** | Weak | Strong | Weak | Moderate | Weak | Weak | (Q1) cannot tell  (Q2) cannot tell  (Q3) Yes | (Q1) Individual  (Q2) Individual  (Q3) Yes  (Q4) Yes | Weak |
| **Bader, 2021** | Weak | Moderate | NA | Weak | Strong | Strong | (Q1) 80-100%  (Q2) cannot tell  (Q3) Yes | (Q1) Individual  (Q2) Individual  (Q3) Yes  (Q4) No | Weak |
| **Eldabea, 2021** | Weak | Strong | Weak | Weak | Weak | Weak | (Q1) cannot tell  (Q2) cannot tell  (Q3) Yes | Q1) Individual  (Q2) Individual  (Q3) Yes  (Q4) Yes | Weak |
| **Thomas et al., 2016** | Weak | Strong | Strong | Weak | Strong | Strong | (Q1) 80-100%  (Q2) Yes  (Q3) Yes | (Q1) Individual  (Q2) Individual  (Q3) Yes  (Q4) Yes | Weak |

# Appendix C: Rating, extracted quotes, and examples of adaptation dimensions for each study

| Study | Language | Person | Metaphor | Content | | Goals | Concept | Method | Context |
| --- | --- | --- | --- | --- | --- | --- | --- | --- | --- |
|  |  |  |  | Assessment tools | Intervention contents |  |  |  |  |
| **Abo-Zaid, 2017** | Assumed to be in Arabic because this study was conducted in GCC country so the Arabic language should not be necessarily explicitly stated |  |  | Researcher translated scales from English to Arabic; reviewed by others, did not pilot it before |  |  |  |  |  |
| **Akras, 2016** | Assumed to be in Arabic because this study was conducted in GCC country so the Arabic language should not be necessarily explicitly stated |  |  | Scale has been validated with Jordan population |  | Agreement on sessions |  | Intervention conducted in a lecture hall at the university.    Intervention was conducted in both group and individual settings to utilise group activities and to have privacy for participants to express their feelings. |  |
| **Al-Ghalib & Salim, 2018** | Assumed to be in Arabic because this study was conducted in GCC country so the Arabic language should not be necessarily explicitly stated |  | "Videos, reading material/tasks given to heighten awareness of emotions, as well as enhance emotional awareness and intelligence to empower spiritual journey to Islam p.148"    Videos on Taqwa and Islamic spirituality | English- version of scales were used. | "Psychological awareness: Feeling/emotion: Videos, reading material/tasks given to heighten awareness of emotions, as well as enhance emotional awareness and intelligence to empower spiritual journey to Islam… p.148”    "Spiritual awareness: Taqwa .. Videos on Taqwa and Islamic spirituality…Divine Power of Dhikr…(chanting) originates in Islamic Spirituality…p.148"    "Silent meditation: Online guided meditation.. They focus mind, heart and soul on God’s presence p.148" |  |  | Intervention was delivered in different formats (in-person, website, WhatsApp) | Discussion of mindfulness in respect of Islamic societies    Discussion of the need of the program to meet Saudi Arabia 2030 Vision and in relation to well-being. |
| **Alhawatmeh et al., 2022** | "...the researcher recorded the intervention instructions in Arabic..p.142" |  |  | Arabic-version were used for all relevant scales.    Explicitly reported that these scales have been validated in previous studies with Arab populations. |  |  |  | Participants accessed reordered intervention based on recommendations.    Duration of intervention was based on previous recommendations for  haemodialysis patients.    Researcher monitored sessions to manage any interruptions. | Discussion about how previous findings cannot be applied to Kuwait populations due to differences in belief systems and self-care practices. |
| **Arnout, 2019a** | Assumed to be in Arabic because this study was conducted in GCC country so the Arabic language should not be necessarily explicitly stated |  |  | Researcher developed the scale to ensure that it is suitable for Arab population; did not pilot it before. |  | Participants contributed to goals setting via discussion    Participants evaluated the program in each session which influenced the next session objectives. |  |  | Discussion of the lack of psychological facilities and support for renal failure patients in Saudi Arabia. |
| **Arnout, 2019b** | Assumed to be in Arabic because this study was conducted in GCC country so the Arabic language should not be necessarily explicitly stated |  |  | Strength personality trait scale was validated before with Saudi students.    Teacher’s psychological well-being scale was developed by the researcher to suit population and culture; did not pilot it before |  | Participants contributed to goals setting via discussion    A discussion with participants in each session to explore their expectations |  | Intervention was delivered in two different settings-in-person and internet-based settings, separately.    Intervention duration and method were set in a way that suit participants’ needs. |  |
| **Al-Shareef, 2020** | Assumed to be in Arabic because this study was conducted in GCC country so the Arabic language should not be necessarily explicitly stated |  |  | Researcher tested the scale with a sample in the same study; however, the method was unclear. |  |  |  |  |  |
| **Al-Sha’rawi, 2021** | Assumed to be in Arabic because this study was conducted in GCC country so the Arabic language should not be necessarily explicitly stated |  |  | Researcher developed the scale; reviewed by experts but did not pilot it before. |  |  |  |  |  |
| **Bader, 2021** | Assumed to be in Arabic because this study was conducted in GCC country so the Arabic language should not be necessarily explicitly stated |  |  | Arabic-version were used for all relevant scales.    Explicitly reported that these scales have been validated in previous studies with Arab populations. |  | A discussion with participants to ensure that their goals and expectation were meeting the main goals of the intervention |  | Original intervention duration was (2 hours, 2 sessions per week, for 16-week) has been modified to (4 hours, 1 session per week, for 16-week) due to participants’ circumstances    Participants were followed-up via WhatsApp, if necessary. | Discussion with participants in which participants highlighted that their current problems are a result of social stress and society negative view. |
| **Eldabea, 2021** | Assumed to be in Arabic because this study was conducted in GCC country so the Arabic language should not be necessarily explicitly stated |  |  | Researcher developed the scale; did not pilot it before. |  |  |  | Intervention delivered via Zoom, WhatsApp group, responded to participants queries and questions at any time |  |
| **Thomas et al., 2016** | All of the written materials the practice audio CDs were in English.  (Information was taken from the qualitative report. |  |  | All relevant scales were presented in both Arabic and English    Stress reactivity measured via DLSS: A culturally grounded tool developed by authors to reflect the UAE citizens’ daily life stressors; piloted before |  |  |  | Intervention arm met during a break in a comfortable seminar before class.    Intervention arm was given accompany materials. | Discussion of burden and associated economic burden of depression for the United Arab Emirates as a developing nation. |

*Note: DLSS= Daily Life Stress Scale; GCC=* *Gulf Cooperation Council.*

# Appendix D: PRISMA checklist

| **Section and Topic** | **Item #** | **Checklist item** | **Location where item is reported** |
| --- | --- | --- | --- |
| **TITLE** | | |  |
| Title | 1 | Identify the report as a systematic review. | p.2 |
| **ABSTRACT** | | |  |
| Abstract | 2 | See the PRISMA 2020 for Abstracts checklist. | p.2 |
| **INTRODUCTION** | | |  |
| Rationale | 3 | Describe the rationale for the review in the context of existing knowledge. | p.2-5 |
| Objectives | 4 | Provide an explicit statement of the objective(s) or question(s) the review addresses. | p.5 |
| **METHODS** | | |  |
| Eligibility criteria | 5 | Specify the inclusion and exclusion criteria for the review and how studies were grouped for the syntheses. | p.5-6 |
| Information sources | 6 | Specify all databases, registers, websites, organisations, reference lists and other sources searched or consulted to identify studies. Specify the date when each source was last searched or consulted. | Main Doc p.6  Sup Doc p.2-5 |
| Search strategy | 7 | Present the full search strategies for all databases, registers and websites, including any filters and limits used. | Main Doc p.6  Sup Doc p.2-5 |
| Selection process | 8 | Specify the methods used to decide whether a study met the inclusion criteria of the review, including how many reviewers screened each record and each report retrieved, whether they worked independently, and if applicable, details of automation tools used in the process. | p.6-8 |
| Data collection process | 9 | Specify the methods used to collect data from reports, including how many reviewers collected data from each report, whether they worked independently, any processes for obtaining or confirming data from study investigators, and if applicable, details of automation tools used in the process. | p.6-8 |
| Data items | 10a | List and define all outcomes for which data were sought. Specify whether all results that were compatible with each outcome domain in each study were sought (e.g. for all measures, time points, analyses), and if not, the methods used to decide which results to collect. | p.6-8 |
|  | 10b | List and define all other variables for which data were sought (e.g. participant and intervention characteristics, funding sources). Describe any assumptions made about any missing or unclear information. | p.6-8 |
| Study risk of bias assessment | 11 | Specify the methods used to assess risk of bias in the included studies, including details of the tool(s) used, how many reviewers assessed each study and whether they worked independently, and if applicable, details of automation tools used in the process. | Main Doc p.7-8  Sup Doc p. 7-8 |
| Effect measures | 12 | Specify for each outcome the effect measure(s) (e.g. risk ratio, mean difference) used in the synthesis or presentation of results. | p.8 |
| Synthesis methods | 13a | Describe the processes used to decide which studies were eligible for each synthesis (e.g. tabulating the study intervention characteristics and comparing against the planned groups for each synthesis (item #5)). | p.6-8 |
|  | 13b | Describe any methods required to prepare the data for presentation or synthesis, such as handling of missing summary statistics, or data conversions. | p.6-8 |
|  | 13c | Describe any methods used to tabulate or visually display results of individual studies and syntheses. | NA |
|  | 13d | Describe any methods used to synthesize results and provide a rationale for the choice(s). If meta-analysis was performed, describe the model(s), method(s) to identify the presence and extent of statistical heterogeneity, and software package(s) used. | p.8 |
|  | 13e | Describe any methods used to explore possible causes of heterogeneity among study results (e.g. subgroup analysis, meta-regression). | NA |
|  | 13f | Describe any sensitivity analyses conducted to assess robustness of the synthesized results. | NA |
| Reporting bias assessment | 14 | Describe any methods used to assess risk of bias due to missing results in a synthesis (arising from reporting biases). | NA |
| Certainty assessment | 15 | Describe any methods used to assess certainty (or confidence) in the body of evidence for an outcome. | NA |
| **RESULTS** | | |  |
| Study selection | 16a | Describe the results of the search and selection process, from the number of records identified in the search to the number of studies included in the review, ideally using a flow diagram. | p. 8  Figure 1 |
|  | 16b | Cite studies that might appear to meet the inclusion criteria, but which were excluded, and explain why they were excluded. | NR |
| Study characteristics | 17 | Cite each included study and present its characteristics. | Table 2, 3, and 4 |
| Risk of bias in studies | 18 | Present assessments of risk of bias for each included study. | Main Doc p.10-11  Sup Doc p.6-7 |
| Results of individual studies | 19 | For all outcomes, present, for each study: (a) summary statistics for each group (where appropriate) and (b) an effect estimate and its precision (e.g. confidence/credible interval), ideally using structured tables or plots. | Table 3 and 4 |
| Results of syntheses | 20a | For each synthesis, briefly summarise the characteristics and risk of bias among contributing studies. | p.8-10 |
|  | 20b | Present results of all statistical syntheses conducted. If meta-analysis was done, present for each the summary estimate and its precision (e.g. confidence/credible interval) and measures of statistical heterogeneity. If comparing groups, describe the direction of the effect. | p. 11-14  Table 2, 3, and 4  Sup Doc p.8-11 |
|  | 20c | Present results of all investigations of possible causes of heterogeneity among study results. | NA |
|  | 20d | Present results of all sensitivity analyses conducted to assess the robustness of the synthesized results. | NA |
| Reporting biases | 21 | Present assessments of risk of bias due to missing results (arising from reporting biases) for each synthesis assessed. | NA |
| Certainty of evidence | 22 | Present assessments of certainty (or confidence) in the body of evidence for each outcome assessed. | NA |
| **DISCUSSION** | | |  |
| Discussion | 23a | Provide a general interpretation of the results in the context of other evidence. | p.14-18 |
|  | 23b | Discuss any limitations of the evidence included in the review. | p.18-20 |
|  | 23c | Discuss any limitations of the review processes used. | p.18-20 |
|  | 23d | Discuss implications of the results for practice, policy, and future research. | p.20 |
| **OTHER INFORMATION** | | |  |
| Registration and protocol | 24a | Provide registration information for the review, including register name and registration number, or state that the review was not registered. | p.21 |
|  | 24b | Indicate where the review protocol can be accessed, or state that a protocol was not prepared. | p.21 |
|  | 24c | Describe and explain any amendments to information provided at registration or in the protocol. | Sup Doc p. 15 |
| Support | 25 | Describe sources of financial or non-financial support for the review, and the role of the funders or sponsors in the review. | p.21 |
| Competing interests | 26 | Declare any competing interests of review authors. | p.21 |
| Availability of data, code and other materials | 27 | Report which of the following are publicly available and where they can be found: template data collection forms; data extracted from included studies; data used for all analyses; analytic code; any other materials used in the review. | Supplementary materials |

*From:*  Page MJ, McKenzie JE, Bossuyt PM, Boutron I, Hoffmann TC, Mulrow CD, et al. The PRISMA 2020 statement: an updated guideline for reporting systematic reviews. BMJ 2021;372:n71. doi: 10.1136/bmj.n71

*Note: Main Doc= main document; NA= not applicable; NR= not reported; P.= page; Sup Doc= supplementary document*

# Appendix E: Protocol amendments

For the content dimension, Bernal and Sáez-Santiago (2006) emphasised that cultural uniqueness should be integrated into all phases of the intervention process in both the assessment and intervention plans. Therefore, the adaptation of assessment tools and intervention content was evaluated separately; consequently, scores on the cultural adaptation scale ranged from 0 to 9 rather than 0 to 8 as what was pre-specified in the protocol.
